# A Tetrahedron-Based Endmember Selection Approach for Urban Impervious Surface Mapping

**DOI:** 10.1371/journal.pone.0093479

**Published:** 2014-06-03

**Authors:** Wei Wang, Xinfeng Yao, Junpeng Zhai, Minhe Ji

**Affiliations:** 1 Key Lab of GIScience, Education Ministry of China, East China Normal University, Shanghai, China; 2 Agricultural Information Institute of Science and Technology, Shanghai Academy of Agricultural Sciences, Shanghai, China; DOE Pacific Northwest National Laboratory, United States of America

## Abstract

The pixel purity index (PPI) and two-dimensional (2-D) scatter plots are two popular techniques for endmember extraction in remote sensing spectral mixture analysis, yet both suffer from one major drawback, that is, the selection of a final set of endmembers has to endure a cumbersome process of iterative visual inspection and human intervention, especially when a spectrally-complex urban scene is involved. Within the conceptual framework of a V-H-L-S (vegetation-high albedo-low albedo-soil) model, which is expanded from the classic V-I-S (vegetation-impervious surface-soil) model, a tetrahedron-based endmember selection approach combined with a multi-objective optimization genetic algorithm (MOGA) was designed to identify urban endmembers from multispectral imagery. The tetrahedron defining the enclosing volume of MNF-transformed pixels in a three-dimensional (3-D) space was algorithmically sought, so that the tetrahedral vertices can ideally match the four components of the adopted model. A case study with Landsat Enhanced Thematic Mapper Plus (ETM+) satellite imagery in Shanghai, China was conducted to verify the validity of the method. The method performance was compared with those of the traditional PPI and 2-D scatter plots approaches. The results indicated that the tetrahedron-based endmember selection approach performed better in both accuracy and ease of identification for urban surface endmembers owing to the 3-D visualization analysis and use of the MOGA.

## Introduction

Urban composition exhibits a high degree of spatial and temporal heterogeneity, which is typically characterized by sharp boundaries and frequent variation of surface materials over space and by their changes mostly as a result of human activities over time [1]. Mapping the dynamics of urban composition, particularly the impervious surface which has emerged as an indicator of urbanization, is essential for understanding human-environment interactions, and hence for better urban planning and management decisions [2], [3].

In recent years, this task has been increasingly performed using remote sensing technology, as it is deemed an effective tool to estimate impervious surface for large areas with relatively low costs and high suitability [4]. Depending on the scale of research, urban impervious surface mapping is conducted with either high and very high resolution satellite imagery (e.g. SPOT, IKONOS, and QuickBird) [5], [6] or the traditional medium-resolution imagery (e.g. the Landsat series) [7], [8]. The high spatial resolution images have the advantages of finer textural details and sharper delineation of ground features, providing a great potential to alleviate the mixed pixel problem. However, new issues related to these image data are found difficult to handle, including noticeable shadows caused by urban topography, such as tall buildings and trees [9], the high spectral variation existing within the same land cover class [10], and the difficulty to obtain a complete cloud-free coverage for large areas in practice [11]. In comparison, the medium-resolution Landsat remote sensors have provided multispectral imagery consistently for more than 30 years, making it a highly stable and reliable source for documenting changes in urban land cover and land use over time [7], [8], [12], [13]. Due to the heterogeneity of urban landscape, however, the application of medium spatial resolution images for urban surface mapping is often limited by the well-known mixed pixel problem [5], [12]. This issue has triggered many studies that are aimed to explore ways of extracting urban surface components at the sub-pixel level [7], [8], [12].

Among many sub-pixel analysis models that were proposed to overcome the mixed pixels problem [3], [8], [11], [14], [15], the V-I-S model developed by Ridd in 1995 is the most widely used one for urban surface components estimation [7], [16]. It simplifies a complex urban environment into a combination of three basic ground components: vegetation, impervious surface, and soil [3]. Much research has successfully applied this model to estimating the composition of urban environments from remote sensing data [2], [4], [12], [17]. In practice, however, V-I-S as a conceptual model may not have a one-to-one match to the spectra of corresponding endmembers. Due to the diversity of materials that comprise urban impervious surfaces, it is almost impossible to develop a single spectral signature or endmember for their representation in the V-I-S model. It is now a general consensus that diverse man-made materials being used for the construction of urban impervious surfaces can be largely modeled into low and high albedo endmember fractions [7], [13]. As a result, numerous applications based on linear spectral mixture analysis (LSMA) have included high albedo, low albedo, vegetation, and soil as the basic four endmembers for urban surface mapping [18], [19]. However, the mismatch between the theoretical basis of the V-I-S model and the modeling practice remains a significant barrier to modeling accuracy improvement, as it can handicap the development of proper tools for endmember identification. Specifically, it is rather awkward to examine a group of transformed spectral data in the 2-D scatter plots for four endmembers that represent three urban surface components, as the current practices commonly do [7], [13], [19]. It is therefore necessary to reexamine the theoretical basis of the V-I-S model and seek possible modification in order to better fit the spectral characteristics of an urban scene. Significant improvement in urban surface mapping should result from a conceptual model that can provide a better description of urban environments [2]. It is also equally important to design a set of proper tools that can provide a better match between model components and their spectral representations during the endmember identification process.

So far the development of practical endmember selection tools for multispectral data has been very slow, as evident in such commercially available software as ENVI and ERDAS Imagine. Most LSMA practices still rely on such conventional methods as pixel purity index (PPI) and 2-D scatter plots of MNF transformed pixels for extracting endmembers from multispectral data. These endmember extraction methods largely depend on manual selection, and the location of and the amount of pixels for an endmember are usually determined via a subjective judgment procedure. Recent efforts for reducing human intervention in endmember selection include the use of artificial intelligence based methods, such as the application of some genetic algorithms to automatically search for optimal solutions in digital image analysis [20], [21]. A noticeable development in this area is the use of a multi-objective optimization genetic algorithm (MOGA) by Saha et al. (2010), who attempted to detect a proper number of spectral clusters by allowing the search to perform over a number of conflicting objective functions [21]. In their application, the random clustering based image segmentation was improved by MOGA, and the selection of cluster centers was intelligent and automatic. MOGA was successfully applied in SVM classifiers to collect a statistically meaningful set of training samples [22]. These studies encourage the application of MOGA to aid endmember extraction.

The main objective of this study was to design a new method which can better implement the four-component conceptual model. This method fits a tetrahedron to the spectral characteristics of urban surface in a 3-D MNF space and provides a multi-objective optimization genetic algorithm to identify the four urban endmembers. The remaining body of this paper is organized as follows. The background of this study, including the common methods of endmember extraction, is presented in the second section, which is followed by a formal proposition of the tetrahedron model and its relevant method for improved urban endmember extraction in the third section. In section 4, the results from applying the new method to the empirical data of Shanghai, China and accuracy comparisons with existing common methods are presented. Section 5 points out the limitation and remaining issues in this research, and section 6 concludes this study. It is hoped that the result of this study may provide useful guidance to improve urban surface component mapping from medium-resolution multispectral data.

### Existing Common Methods for Endmember Extraction: A Background

Urban surface components mapping based on the V-I-S model involves four steps: image preprocessing, endmember selection, spectral mixture analysis modeling, and generation of fraction images. The key to an accurate urban surface mapping is to find an appropriate suite of endmembers [23]. Endmembers can be either selected from pre-constructed spectral libraries (built from field or laboratory measurements) [24], derived from image data themselves [7], or generated from the integration of relevant library and image endmembers [25]. In practice, image based endmembers are often used because their spectra are easy to obtain and they are measured at the same scale as the image data [26]. Several well-developed approaches relying on spectral features for image-based endmember extraction include two-dimension (2-D) scatter plots [27], pixel purity index (PPI) [28], N-FINDR [29], and automated morphological endmember extraction (AMEE) [30]. Among these methods, 2-D scatter plots and PPI are most widely used because of their availability in many remote sensing software packages (e.g. ENVI) and their satisfactory results [13], [17]. These two methods will be used for comparisons in this study and described in more detail as follows.

### 2-D scatter plots

**Figure 1:**
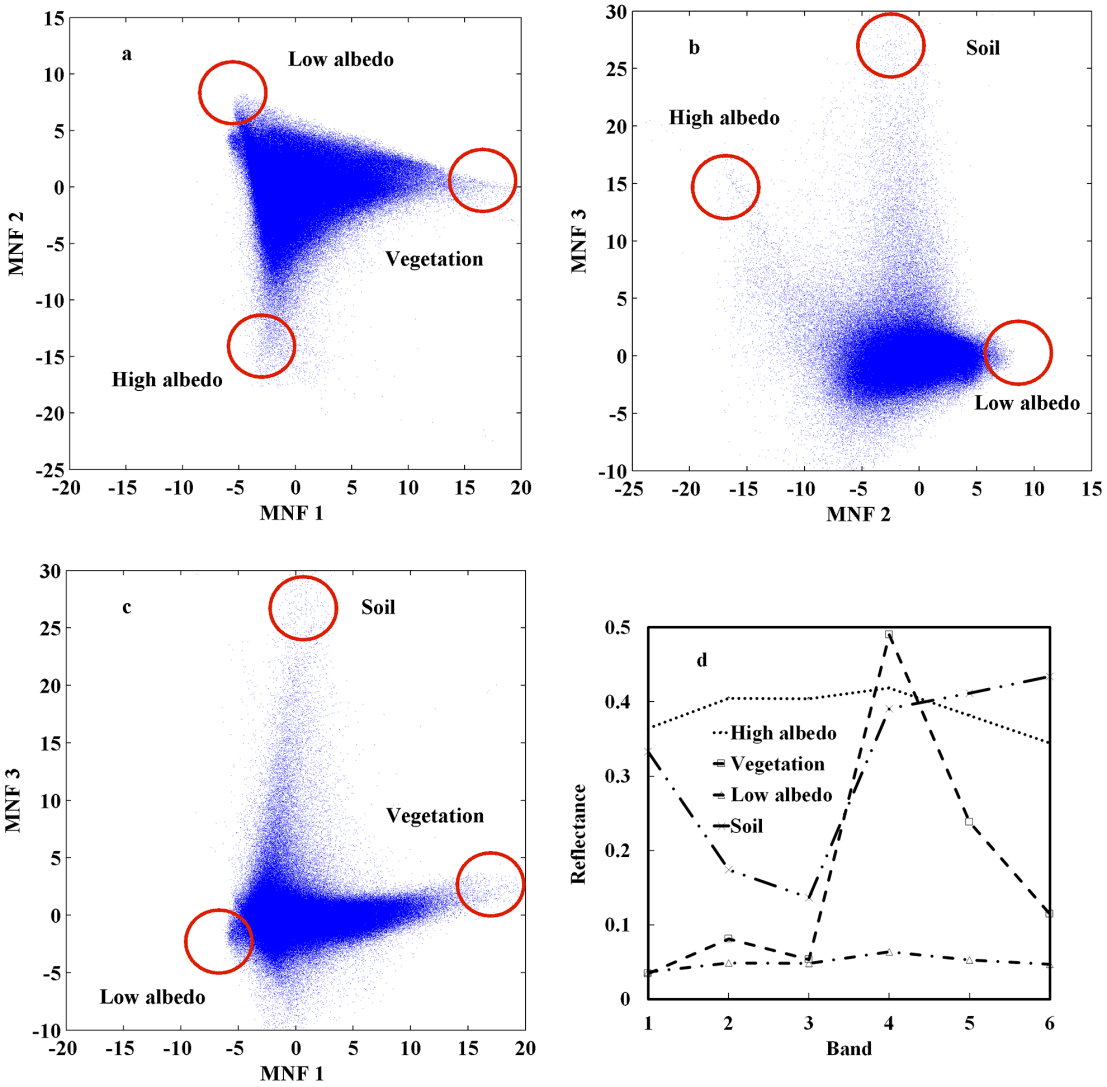
Endmember extraction by 2-D scatter plots method. (a–c) 2-D scatter plots of MNF showing the locations of potential endmembers; (d) Spectral reflectance curves of the endmember pixels selected via MNF 2-D scatter plots are plotted against the original bands.

In urban remote sensing, a 2-D scatter plot is commonly used to visualize the distribution of pixel spectra in either original or transformed manner for endmember identification. In the case of original spectra, for example, the red and NIR data are deemed a good combination in the scatter plot visualization to uncover potential endmembers [31]. Because of the well-known between-band collinearity, however, an orthogonal transform is usually applied before scatter plot visualization, such as principal component analysis (PCA) [27] and minimum noise fraction (MNF) [12], [32]. The effect of an orthogonal transform is to remove inherent data redundancy so that groups of pixels representing individual endmembers in the image can be clearly defined as vertices in the scatter plot. The 2-D scatter plots in Figure 1, for instance, exhibit the data distribution of the first three MNF components for the Landsat ETM+ image to be used in this study. The extreme values located at the vertices of the shape are deemed to belong to respective endmembers of the image. These endmember pixels are further traced back to the original image for their biophysical meanings in terms of land cover types. Then, the spectra of the pixels belonging to an endmember are averaged to represent the final spectra of that endmember (Figure 1d).

**Figure 2:**
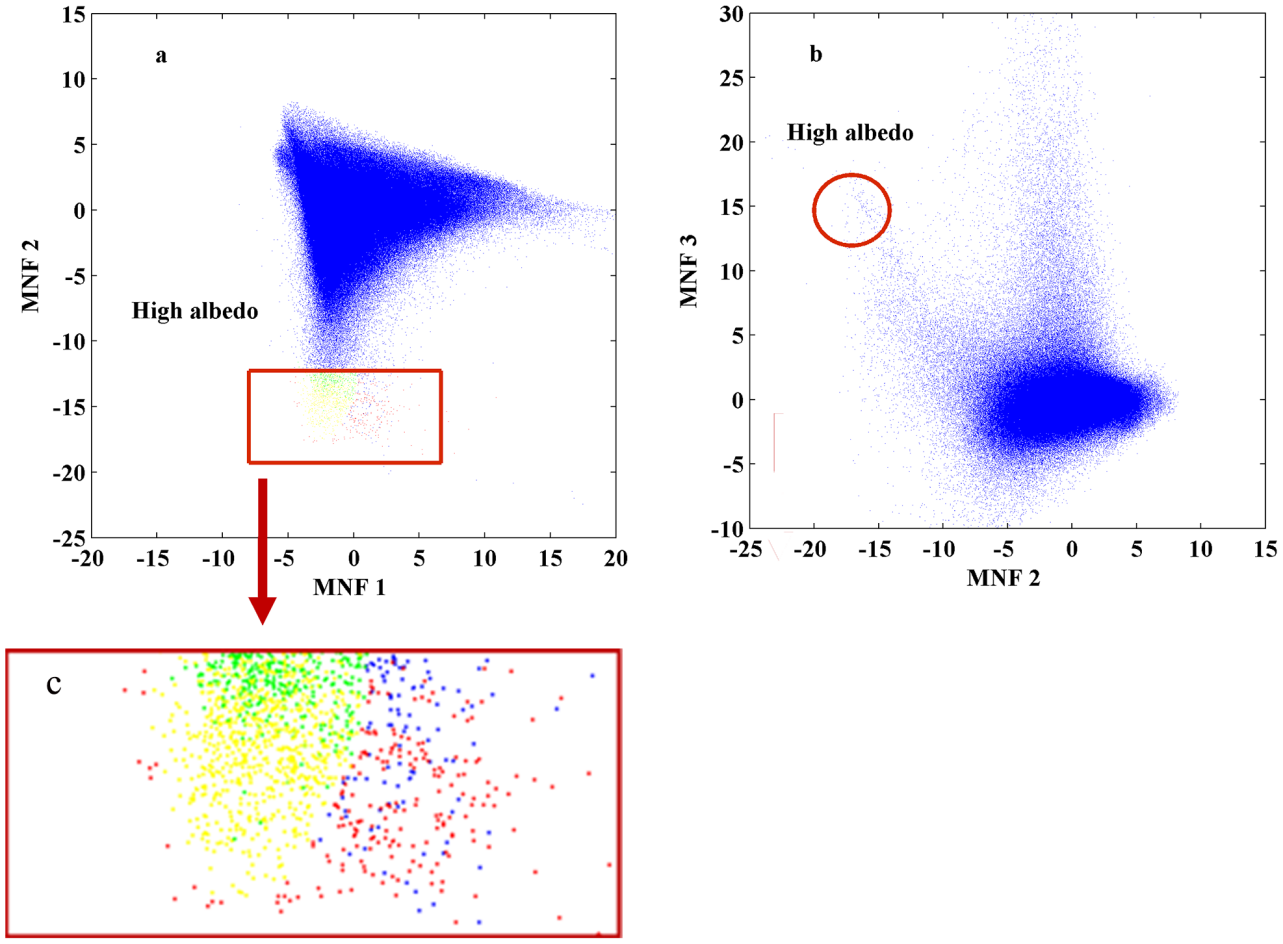
Comparison of high albedo endmembers selected from different 2-D MNF scatter plots of MNF. (a) Overlaying those endmembers derived from MNF 2&3 (b) with those from MNF 1&2, reveals mismatched cases: green points, representing the MNF 1&2 induced high albedo pixels that are not present in MNF 2&3, and red points, representing the opposite cases. The yellow points represent the endmember pixels existing on both MNF 1&2 and MNF 2&3; (c) The overlay of high albedo endmembers is zoomed up in the red rectangular box.

Although the 2-D scatter plots method is widely utilized in many studies [7], [17], [19], [33], the inherent 2-D viewing scheme severely limits its ability to reveal endmember pixels for the four-component model. Specifically, a 2-D scatter plot can best exhibit the separation of no more than three endmember vertices, as shown in Figure 1a, b, and c. Since it is often impossible to examine all four endmembers simultaneously, multiple scatter plots must be viewed side by side so that a complete picture can be generated. This practice makes it rather difficult to select the same pixels for an endmember from different scatter plots. For example, it seems that the high albedo endmember in Figure 1 can both be determined by MNF 1&2 and MNF 2&3, yet the overlay of the two results (Figure 2a and 2c) reveals a significant difference. While there exists a large group of concurrent pixels (colored in yellow), the number of identified pixels that do not coexist on both plots is also very large (green for pixels only from MNF1 & MNF2, red for pixels only from MNF2 & MNF3). The preliminary analysis indicates that the 2-D scatter plots method can fail to provide sufficient stability and reliability in the selection of endmembers, which will inevitably affect the quality of urban surface mapping. As a result, the accuracy of urban surface decomposition heavily depends on prior knowledge and skills of the analyst involved in the visual selection of endmembers.

### Pixel purity index

Ever since its initial proposal and development by Boardman et al. (1995) [28], the pixel purity index (PPI) has been widely used for endmember extraction because of its successful application and availability in the ENVI software. The working principle of PPI is as follows: for each pixel an index value is generated by repeatedly projecting pixel into a set of randomly directed vectors, and a cutoff index value is chosen as a threshold to delineate pure pixels. Then, the pure pixel spectra are loaded into the N-dimensional visualizer in ENVI for further manual selection. It selects a set of vertices of a convex hull in the N-dimensional space, which are supposed to be pure signatures (endmember) for the image. This step requires a significant degree of human intervention from an experienced operator [34]. PPI is sensitive to the input parameters: the number of vectors and the threshold of cutoff index value [34], [35], but there is no theoretical guidance on how to set these parameters, just relying on user experience. In addition, the vectors are randomly generated, so the repeatability of the results is poor.

**Figure 3:**
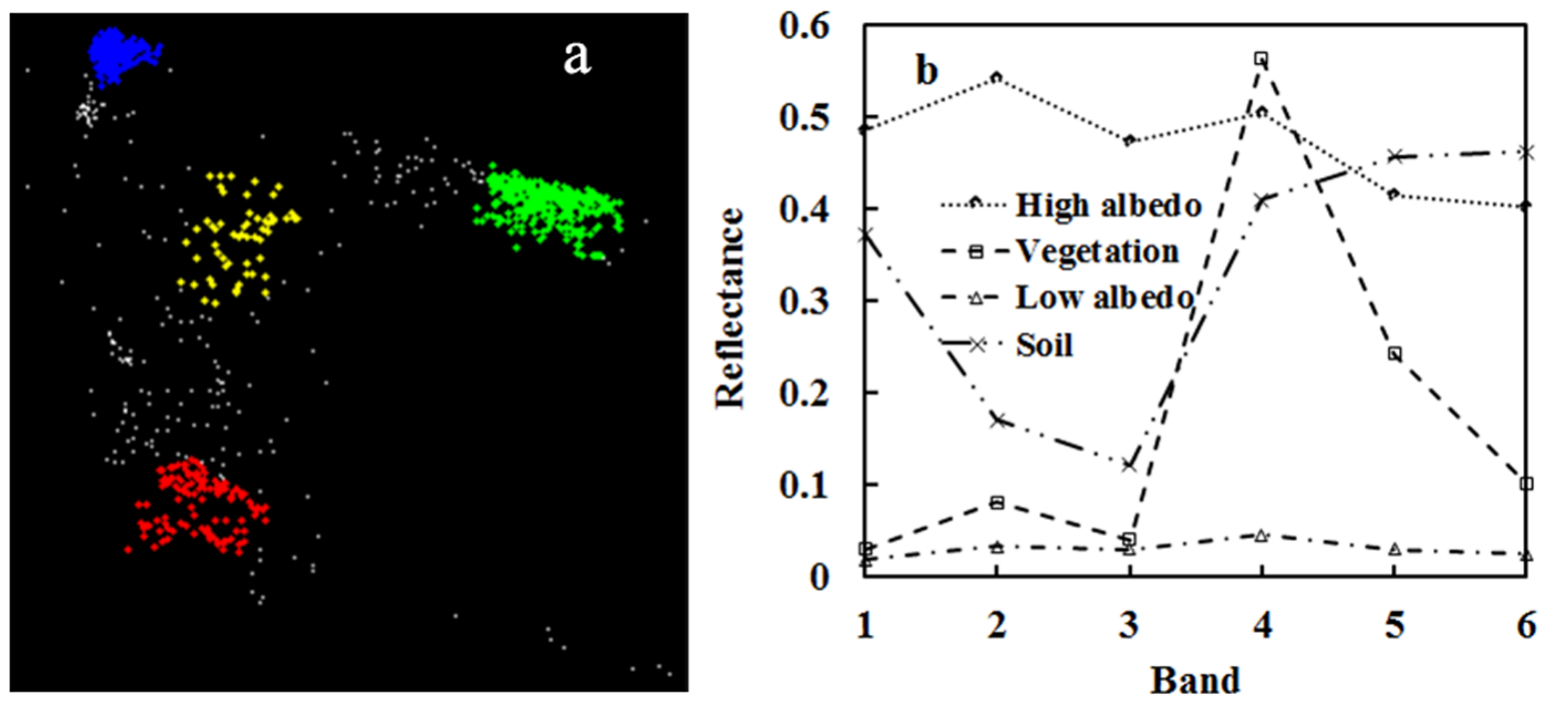
Selected endmembers by the PPI method. (a) endmembers displayed in different colors (red - high albedo, green - vegetation, blue - low albedo, and yellow - soil); (b) their spectral reflectance characteristics as summarized from their corresponding image pixels.

Figure 3 shows the PPI-based selection of the four urban endmembers from the data of this study. The N-dimensional visualizer allows for free rotation of the MNF space to view pure pixels, but it is not an easy tool for an inexperienced user to operate. Since the visualizer only allows displaying of the selected pixels without a geometrical shape that can be formed with the full set of pixel data, it shows no obvious distribution pattern as a framework of reference for pixel viewing. Instead, the selection of true pure pixels has to depend on further clustering of the selected data points and interactive comparison with their corresponding features on the original image.

## Methodology

### The V-H-L-S model and its tetrahedron representation

**Figure 4:**
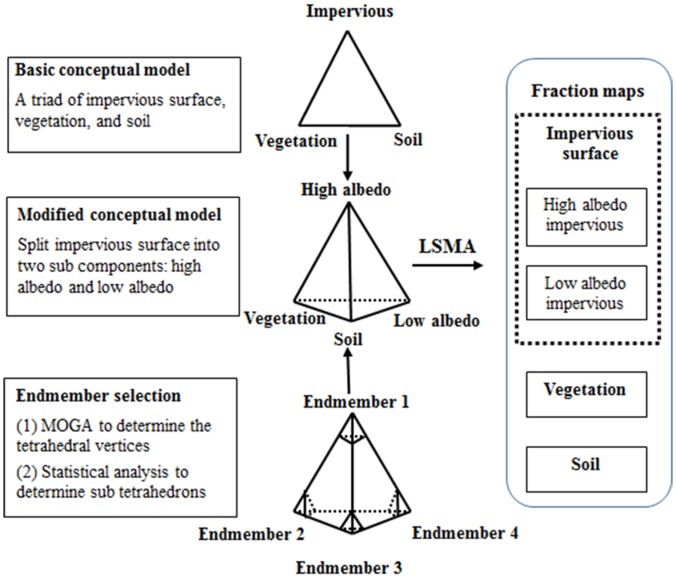
Flowchart of the tetrahedron-based endmember selection approach.

As modified from the classic V-I-S model, the V-H-L-S model divides the impervious surface component into two sub components, i.e. high albedo and low albedo, so that they can be made in accordance with the spectral characteristics of impervious materials with broad differences. The conceptual framework of the tetrahedron-based urban endmember selection and fraction mapping is summarized in Figure 4.

**Figure 5:**
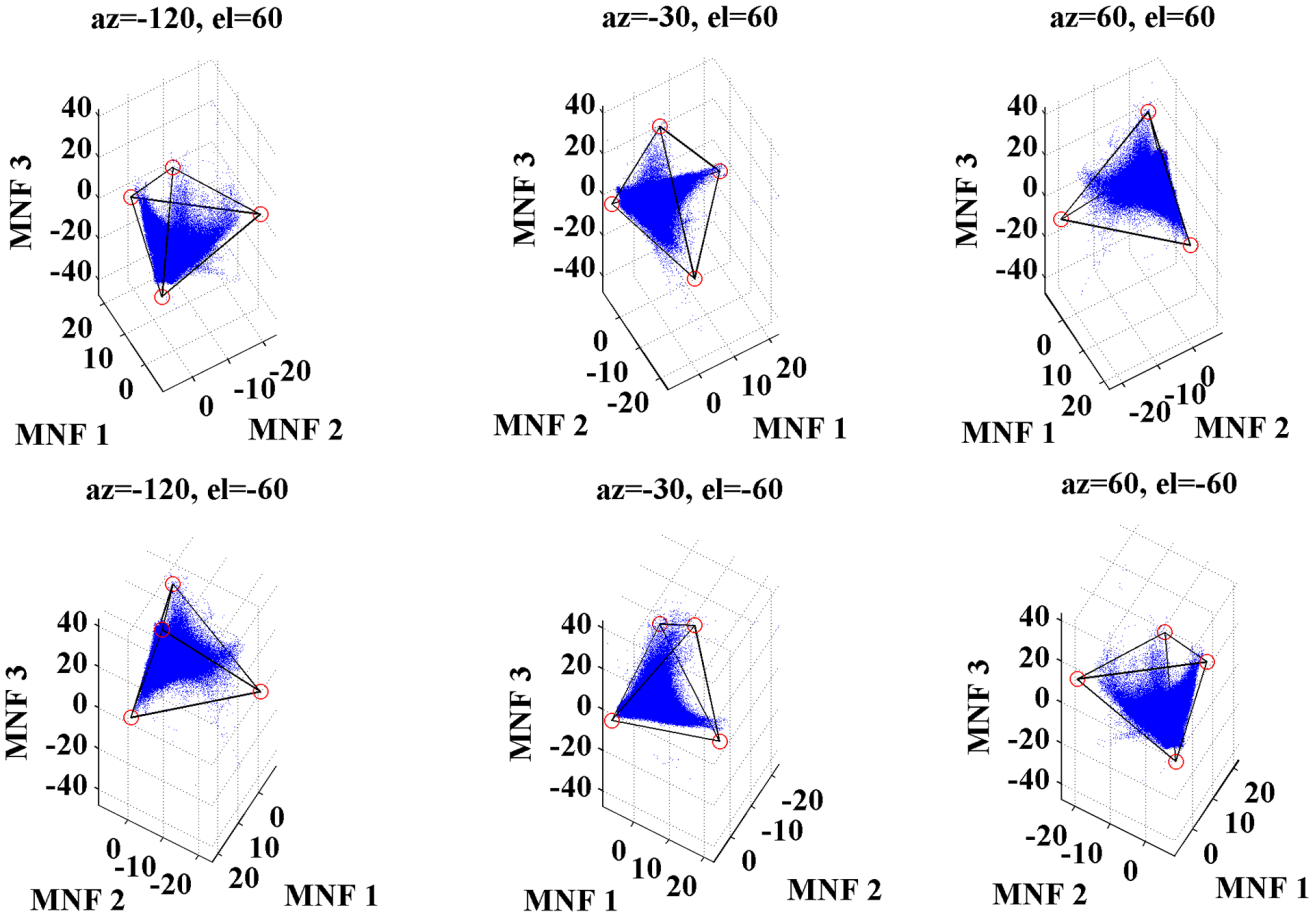
A 3-D scatter plot of MNF transformed pixels viewed from different angles (a shape of distribution approximates to a tetrahedron, with most pixels being enclosed inside the solid).

The V-H-L-S spectral unmixing procedure is hereby implemented in a 3-D MNF transformed feature space. As illustrated in Figure 5, the actual distribution of transformed pixels in the 3-D space is approximated by a hypothetical tetrahedron, with pure pixels located around the vertices and mixed pixels contained in the main body. Viewing the 3-D scatter plot from different angles will help evaluate the “endmembership” of each pixel based on all four components simultaneously. The adoption of a tetrahedron can best exploit the geometry of the solid for the MNF-based model representation, i.e. the four vertices of the tetrahedron may well coincide with pixels of extreme spectral values located at the corners of the three-dimensional distribution. This way the endmember selection is no longer limited by the 2-D view as the traditional scatter plot offers, and proper interactive visual graph tools, such as rotate, zoom, and pan, can be implemented to assist precise locating of pure pixels and minimize identification errors.

### Endmember selection

The V-H-L-S model based endmember selection procedure consists of two key phases: determination of the tetrahedral vertices and selection of potential pure pixels located around the vertices. The task in the first phase is to search, among all pixel combinations in the 3-D space, a group of four vertices that can define an optimal tetrahedron for endmember selection. The criteria for optimum are two-fold, i.e. the tetrahedron so formed must contain the maximum number of pixels in a shape of minimum volume [36]. In this study, the vertices of the optimal tetrahedron were determined via a multi-objective optimization genetic algorithm (MOGA), a method typically utilized in the situation where simultaneous satisfaction of multiple conflicting objectives (such as volume and number in this study) is required [37]. In contrast to traditional multi-objective optimization methods, the MOGA does not combine the individual objective functions into a single objective function, which can avoid the selection of the proper weights for each objective function to characterize the decision-maker's preferences. In practice, it can be very difficult to precisely and accurately select these weights, and small perturbations in the weights can sometimes generate different solutions [38]. Unlike traditional multi-objective optimization methods, MOGA optimizes all the objectives separately and simultaneously.

The MOGA problem in this study can be formulated into the following equation:
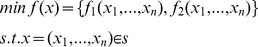
(1)


In eq. (1), *f*(*x*) is a multi-objective function with two objectives: *f*_1_(*x*) and *f*_2_(*x*). *f*_1_(*x*) is the volume of tetrahedron to be minimized in the function. *x_1_,…,x_n_* represents the coordinates of four vertices of the tetrahedron in the 3-D space, and hereby *n* = 12. *s* denotes the solution space, which may be roughly defined in a given range depending on the distribution of points in 3-D space. The range is the Euclidean distance specified in the spectral unit and serves as a data volume constraint in MOGA for the purpose of computational efficiency. Suppose *a_1_*(*x_1_, x_2_, x_3_*), *a_2_*(*x_4_, x_5_, x_6_*), *a_3_*(*x_7_, x_8_, x_9_*), and *a_4_*(*x_10_, x_11_, x_12_*) are four independent points in 3-D space, the volume of the tetrahedron *f*_1_(*x*) formed by these points is then determined as follows:
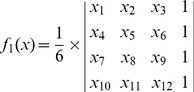
(2)
*f*_2_(*x*) represents the number of pixels within the tetrahedron to be maximized for our purpose. In eq. (1), the negation of *f*_2_(*x*) is used to make it in line with *f*_1_(*x*) for the operation of multi-objective minimization. Function *f*_2_(*x*) results from a process of accumulating points that are identified as being located within the space formed by the four planes of the tetrahedron. The identification rules are expressed as a set of plane equations. Suppose vertices (*a*_1_, *a*_2_, *a*_3_) define plane *A*_1_ with equation (*ax*+*by*+*cz*+*d* = 0) in 3-D space, and coefficients (*a*, *b*, *c*, *d*) are determined by solving the plane equation according to the known points (*a*_1_, *a*_2_, *a*_3_). To judge whether any pixel *p*(*x*, *y*, *z*) is in plane *A*_1_, a fourth vertex of the tetrahedron, *a*_4_, is involved in the following computation: 
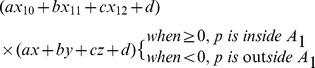
(3)


The meaning of eq. (3) states that only and if only plane equation *A*_1_ has the same sign as the fourth vertex, can pixel *p* be enclosed in plane *A*_1_ of the tetrahedron. This evaluation process is repeated to judge the relationship of *p* with the other three tetrahedral planes in 3-D space. Therefore, *p* is considered a pixel in the tetrahedron only if it is inside the four planes of the tetrahedron simultaneously.

Compared with its single-objective counterpart, the solution of MOGA is not a single point, because the two-objective optimization is rather complicated as the objectives may conflict with each other. A set of vertices that enclose the maximal pixels may not yield a tetrahedron of the minimal volume. To deal with this conflict, the Pareto front solution was adopted, that is an effective means to provide the trade-off between required multi-objectives [39]. MOGA would return a set of Pareto optimal solution, each of which satisfies the objectives at an acceptable level without being dominated by any other solution [38], [39]. Each Pareto optimal solution is optimal in the sense that there is no other solution that can improve at least one of the objectives without degrading any other objectives [39]. All solutions in a Pareto set will provide useful and flexible decision-making information to assist a user to find the most optimal solution [38]. The designer can then choose from this set of solutions according to the relative satisfaction and preference of the problem [40]. In this work, an interactive Post-Pareto analysis is taken to aid decision makers in finding the best solution from the resulting Pareto set [40], [41]. The best solution is defined by the L_2_ norm [41]. The method minimizes the geometric distance between Pareto set solution and an ideal solution (called “Utopia point” in the L_2_ norm definition), according to the following formula:
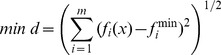
(4)where 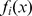 is the value of multi-objective function i for each solution in the Pareto set, and m is equal to 2 in this work. 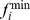 is the “Utopia point” that can be achieved theoretically, and is practically computed by the minimum value of each multi-objective function in the Pareto set [40], [42].

The second phase of endmember selection starts from the completion of identifying the optimal tetrahedron, of which the four vertices in the 3-D MNF space serve as the locations of endmembers in the V-H-L-S model. It is assumed that the data points close to the vertices are representative of the endmembers. Pure pixels for each endmember are then delineated using a sub tetrahedron (Figure 4) having its apex coinciding with a vertex of the optimal tetrahedron, its base paralleled to the facet opposite of the vertex, and its height interactively determined by the corresponding spectral features on the image. The interactive approach starts with a statistically acceptable number of points (e.g. 30) and moves the base progressively away from the apex with a given height increment as 0.1% of the total height of the tetrahedron. In each increment, the spectral characteristics of the enclosed pixels are computed and compared with those from the previous increment. The height determination process stops when the comparison yields no significant difference.

### Linear spectral mixture analysis

Linear spectral mixture analysis (LSMA) is an effective approach to mapping urban surface material fractions at the sub-pixel level [7], [12]. The method assumes the existence of a linear combination of land cover types within the spectral signature of each pixel in a remote sensing image. The fraction of each land cover type, known as an endmember when spectrally defined, can be calculated by modeling the linear relationship between the mixed pixel spectra and the spectra of the endmembers. Formally defined, the LSMA model for unmixing per-pixel spectra into fractional abundances takes the following form: 
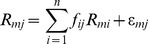
(5)where *i* = 1, …, *n*, *n* is the number of endmembers; *R_mj_* is the reflectance for each band *m* in each pixel *j*; *R_mi_* is the spectral reflectance of band *m* of endmember *i*; *ε_mj_* is the error for band *m* in each pixel *j*; *f_ij_* is the fraction of endmember *i* in each pixel *j*; For a constrained unmixing solution, *f_ij_* is subject to the following restrictions: the fractions are non-negative, and they sum to one.

### Accuracy assessment

In this study, the tetrahedron-based endmember selection approach is evaluated against the traditional 2-D scatter plots and PPI methods in three aspects. The first aspect is concerned with the model residuals, which serve to indicate the representativeness of the selected endmembers for the spectra of mixed pixels. The root mean square error (*RMSE*) of a fraction map, formally expressed below, is employed as a summarized error indicator to be used for cross-method comparisons. 
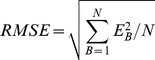
(6)where 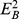 is the residual of the linear least squares model for band *B*, and *N* is the total number of bands.

The second evaluation involves comparing the estimated impervious fractions to their corresponding “ground truth” sampled from low-altitude color-infrared aerial photographs. A total number of 150 samples were selected using the stratified random sampling method, with each sample covering a ground dimension of 90×90 m. Three error indicators, including *RMSE*, mean absolute error (*MAE*), and the coefficient of determination (*R^2^*), were calculated to access the accuracy of impervious surface estimates [43], [44]. *MAE* is the average absolute value of the difference between predicted and observed fraction values. In an ideal case, both *RMSE* and *MAE* should equal zero, and the *R^2^* value would approach one. Besides, the 1∶1 scatter plot of estimated versus actual impervious surface fraction is drawn to reveal the performance of the models. The *RMSE* and *MAE* are formally defined as:
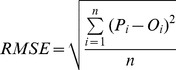
(7)

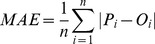
(8)where *P_i_* is the estimated impervious surface fraction for sample *i*; *O_i_* is the actual impervious surface fraction obtained from the aerial photographs for sample *i*; and *n* is the total number of samples.

In the third evaluation, visual comparisons are made between the V-H-L-S induced impervious surface fraction map and the low-altitude color-infrared aerial photographs for four selected urban regions, each representing medium-density residential, high-density residential, very-high-density residential, and commercial land use types, respectively. Due to the fact that the low-density residential was nearly absent within the coverage of the acquired aerial photos, this land use category was neglected from this study.

All aforementioned image unmixing and statistical analysis were implemented in the MATLAB 2012a environment.

## A Case Study Application

### Study area and experimental datasets

**Figure 6:**
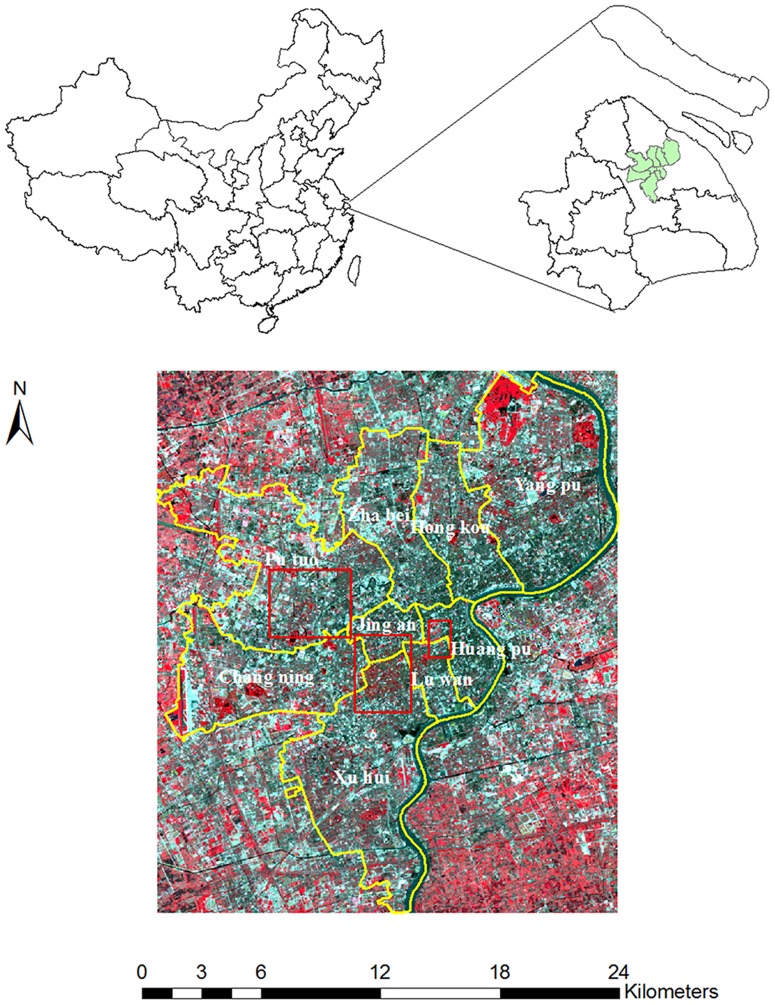
Study area located in the urban core of Shanghai and shown on the false-color composite image of Landsat ETM+ multispectral data, acquired on 3 July 2001 (The red box delineates the coverage of the color-infrared aerial photographs).

The study area is the main urban portion of Shanghai, a megalopolis situating at the east coast of China and ranging between 31°32′N-31°27′N latitude and 120°52′E-121°45′E longitude. It mainly consists of nine administrative districts (i.e. Zhabei, Jing'an, Hongkou, Yangpu, Changning, Xuhui, Luwan, Jiading, and Putuo) (Figure 6) and includes built structures from different development stages. The city had experienced its first rapid expansion during the late 1990s, and this trend had continued into the new century and later reached its climax before the World Expo in 2008. Much of the agricultural land in the inner city has now been turned into residential and commercial land uses as well as paved infrastructure. The diversity of built structures, old and new, makes the urban core of the city an ideal test ground for impervious surface estimation. Mapping the composition of urban environments is crucial to urban planning and sustainable development in Shanghai.

The primary spectral data is a Landsat 7 Enhanced Thematic Mapper plus (ETM+) image (path 118/row 38) covering the main urban portion of Shanghai. The data was acquired on 3 July, 2001 under clear weather conditions. When obtained from the USGS Earth Resource Observation Systems Data Center, the image was found to be already preprocessed for radiometric and geometrical correction to the 1G quality level. For the purpose of this study, the image was further rectified to the Universal Transverse Mercator (UTM) coordinate system. To further remove any possible atmospheric effect, the digital numbers (DN) of the image were converted to at-sensor radiance (top-of-atmosphere radiance) using the published ETM+ calibration constants, and the FLAASH atmospheric correction model was applied to transform spectral radiance to spectral reflectance.

For the purpose of model validation and accuracy assessment, the study also collected a set of color-infrared aerial photographs, acquired during March and April of 2000, to be used as reference data. These digital aerial photos possessed a 0.6-m spatial resolution and were spatially registered to the rectified ETM+ data for further analysis.

Since water bodies spectrally resemble low albedo features and thus interfere the extraction of impervious surfaces, it is a common practice to remove them from the ETM+ scene before further analysis (Wu, 2004). This study employed the following modified normalized difference water index (MNDWI) [45] for water mask generation. 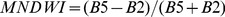
(9)where *B2* and *B5* are the 2nd (green) band and the 5th (mid-infrared) band of the ETM+ image, respectively. The water mask was generated from the index values using an empirically determined threshold. All image preprocessing operations were performed on the ENVI 5.0 image processing platform.

### Extraction of endmembers

**Figure 7:**
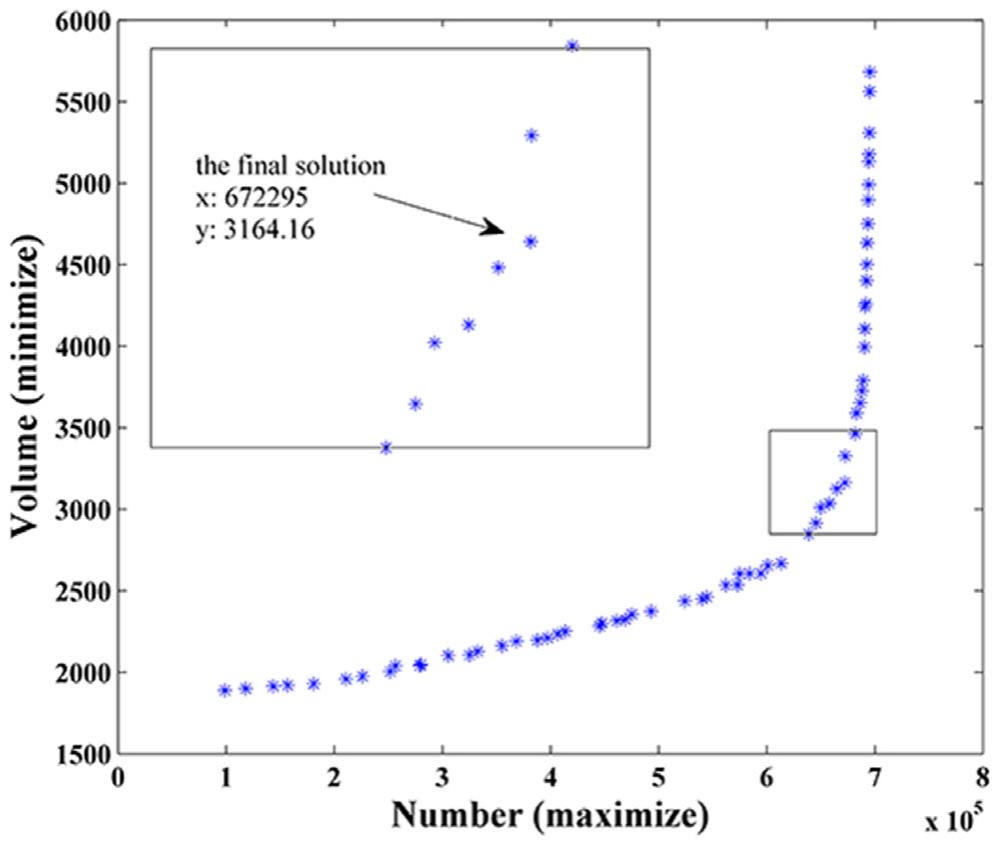
Pareto optimal solutions found by the proposed algorithm.

Based on the V-H-L-S framework outlined in section 3.1, a set of potential solutions using MOGA were obtained and shown in Figure 7. With the increase of the number of the transformed pixels within the tetrahedron, the volume increase started at a low speed first and then accelerated. Among these Pareto set solutions, the solution with a volume of 3164.16 and a pixel count of 672295 (more than 95% of the total pixels in the data cloud) had the minimal L_2_ norm value, and it was selected as the final best solution. It situates at the turning point of the Pareto Front where the slope significantly increased (Figure 7). An optimal trade-off between volume and count was achieved, which ensured a compact distribution of pixels within the tetrahedron.

**Figure 8:**
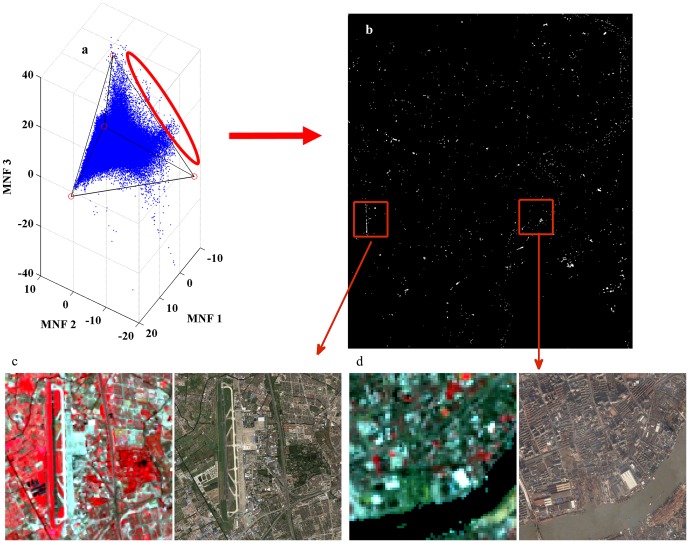
The distribution of ETM+ pixels in a 3-D MNF transformed space. (a) their optimal tetrahedron with vertices in red; (b) the spatial location of the outlying pixels circled by the red ellipse; (c and d) compared to the original ETM+ image and the high-resolution image from Google Earth for their physical identities.

As a necessary quality control step, the evaluation of the pixels unenclosed within the optimal tetrahedron (Figure 8a) was conducted. These pixels (Figure 8b) were checked visually against their corresponding ground features on both the original ETM+ image and the high-resolution Google Earth image (Figure 8c and d). The comparison indicated that these pixels were spectral outliners. The pixels inside the red ellipse in Figure 8a, for example, mostly corresponded to those ground features that have peculiar or extreme spectral characteristics. The linear feature located near the left edge of Figure 8b, for instance, was found to be the airport runways (Figure 8c), implying its use of unique pavement materials. Other outlying pixels matched the roof of some buildings showing a marked difference in spectra from those in the surrounding, such as the China Shipping Pavilion (Figure 8d). As part of urban impervious surface, these outliers can typically be handled with other such techniques as density slicing or decision trees [8].

**Figure 9:**
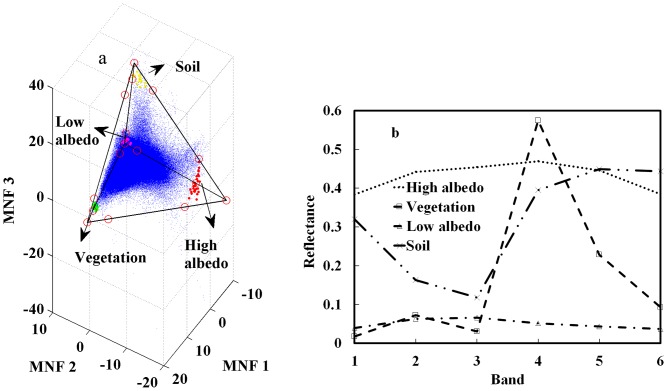
The locations and spectral reflectance characteristics of potential endmembers in the 3-D MNF scatter plot. (a) the vertices of the sub tetrahedron were visually identified and denoted as red hollow points, and pixels belonging to each endmember were confined within a sub tetrahedrons at the vertices of the optimal tetrahedron, displayed as green (vegetation), red (high albedo impervious surface), pink (low albedo impervious surface) and yellow (soil) solid points; (b) spectral reflectance characteristics of the selected endmembers were charted for further analysis.

The next step involved identification of pure pixels for endmember extraction using the method introduced in section 3.2. Pure pixels were defined as those within the limit of individual sub tetrahedrons, each being located at one of the four vertices of the optimal tetrahedron. The height (denoted as red hollow points in Figure 9a) of each sub tetrahedron was interactively determined through the comparison of the candidate endmember pixels between the 3-D scatter plot and the corresponding features on the ETM+ image. The pixels in each sub tetrahedron were symbolized in Figure 9a with a different color to represent their belonging endmember, i.e. green for vegetation, red for high albedo impervious surface, pink for low albedo impervious surface, and yellow for soil. The original spectra of all four extracted endmembers were charted as spectral reflectance curves (Figure 9b) for further analysis.

**Figure 10:**
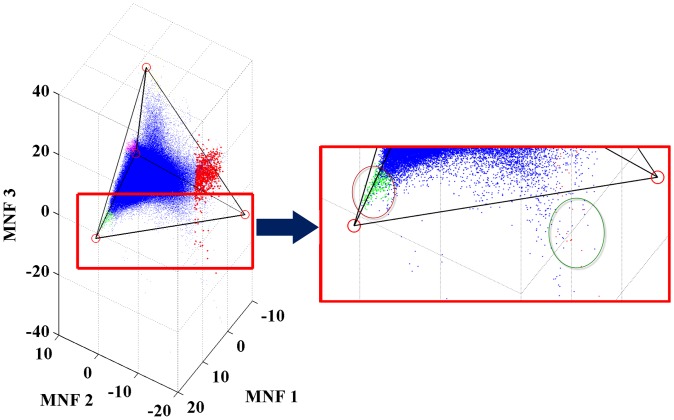
Endmember pixels obtained through 2-D scatter plots are displayed in 3-D space (green – vegetation, red – high albedo, pink – low albedo, yellow – soil). Some pixels in the sub tetrahedron were not selected as endmember pixels in 2-D space (see red circle), whereas some endmember pixels determined by 2-D scatter plots were outside the sub tetrahedron (see green circle).

It is interesting to compare the above results to the endmember pixels derived using the 2-D scatter plots approach. By displaying the 2-D results in the 3-D tetrahedron space, both commission and omission were readily seen (Figure 10). Some vegetation pixels (colored in green) within the sub tetrahedron, for instance, were found not among those selected using the 2-D scatter plots. The opposite also exited, as a portion of pixels labeled as high albedo impervious surface (colored in red) in 2-D scatter plots were found outside its corresponding sub tetrahedron (detailed in Figure 10b). It thus clearly demonstrated that projecting candidate pixels from 3-D space to 2-D space changed the distribution pattern, which was likely to cause misinterpretation during the pure pixel delineation and in turn decrease the accuracy of endmember selection.

### Generation of fraction maps via LSMA

**Figure 11:**
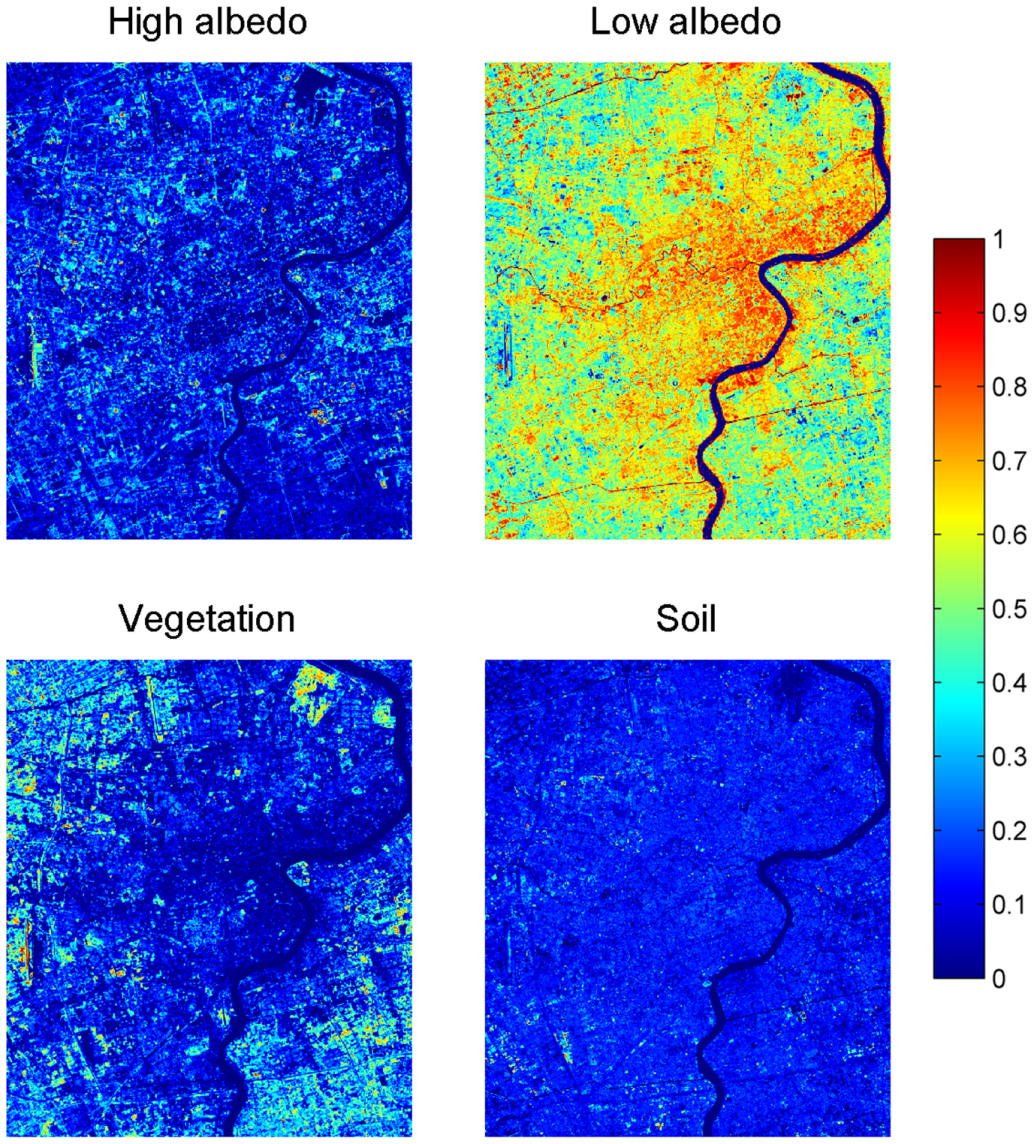
Four endmember fraction maps resulting from LSMA results with endmembers identified from the tetrahedron-based endmember selection approach (fraction values range from 0 to 1, with the lowest values in blue and the highest values in red).

The fraction maps resulting from the application of LSMA with the four endmembers derived using the optimal tetrahedron approach were illustrated in Figure 11. Visually, these maps were found to reflect the generic distribution pattern of concerned features on the ground. The high albedo impervious surface fractions are mainly related to impervious materials with high reflectance values in all spectral bands, such as the roof of new buildings and large commercial structures that mainly concentrate in the new development zone. The low albedo type of impervious surfaces is mainly comprised of old buildings and industrial areas from the early development in the city, which dominate the west of Huangpu River and along Suzhou River. Vegetation fractions increase markedly from close to none at the urban center to medium or high levels in the urban fringe, and high fractions are mostly related to healthy grass and trees (green land and parks in urban areas) and crops in agricultural fields. The soil fractions are far less dominant in any part of the city, only with some isolated patches and spots signifying the result of concurrent farming practices in the rural area and construction activities inside the city.

**Figure 12:**
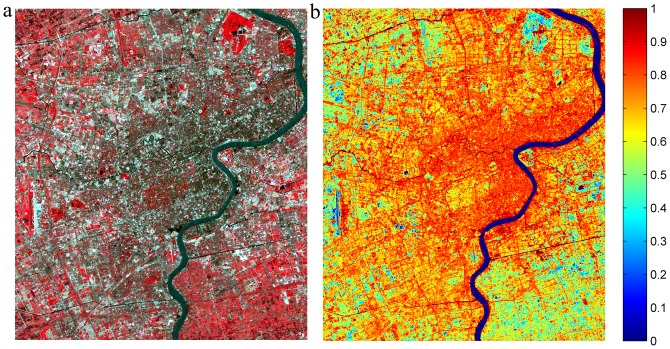
Distribution of Shanghai impervious surface. (a) the original Landsat ETM+ imagery; (b) the fraction map derived with the V-H-L-S model.

Different from vegetation and soil that are singularly defined during spectral endmember extraction, the dual-endmember representation of impervious surface requires that the fraction maps of high and low albedos be combined in order to achieve a complete picture. Compared to the original ETM+ image (Figure 12a), the combined fraction map (Figure 12b) seemed to match the common sense about the urban impervious surfaces in Shanghai: high fraction values located in commercial and high-density residential areas as well as transportation networks, medium values in the medium-density residential areas, and lower values in suburbs and vegetated areas.

### Evaluation of urban impervious surface derivation

As discussed in section 3.4, the tetrahedron-based endmember selection method is evaluated from three perspectives. The goodness of fit of the new method was first evaluated over the traditional 2-D scatter plots and PPI methods via comparative analysis of their respective LSMA model residuals. The 2-D scatter plots and PPI based endmembers were hence created following their usual procedures, yielding two sets of fraction maps that are not shown in the article due to their high visual similarity with maps from the tetrahedron-based method. Instead, the comparison was performed quantitatively with the measure of *RMSE* (see eq. (6)) between the observed and predicted values, hereby aiming to evaluate the overall accuracy of an impervious surface fraction map resulting from the LSMA model. For all three endmember extraction methods involved in the analysis, the *RMSE* of the entire map was 0.014 for the 3-D method, 0.016 for 2-D scatter plots, and 0.021 for PPI. Thus, the error level was rather low for all three methods, and the between-method difference was also minimal.

**Figure 13:**
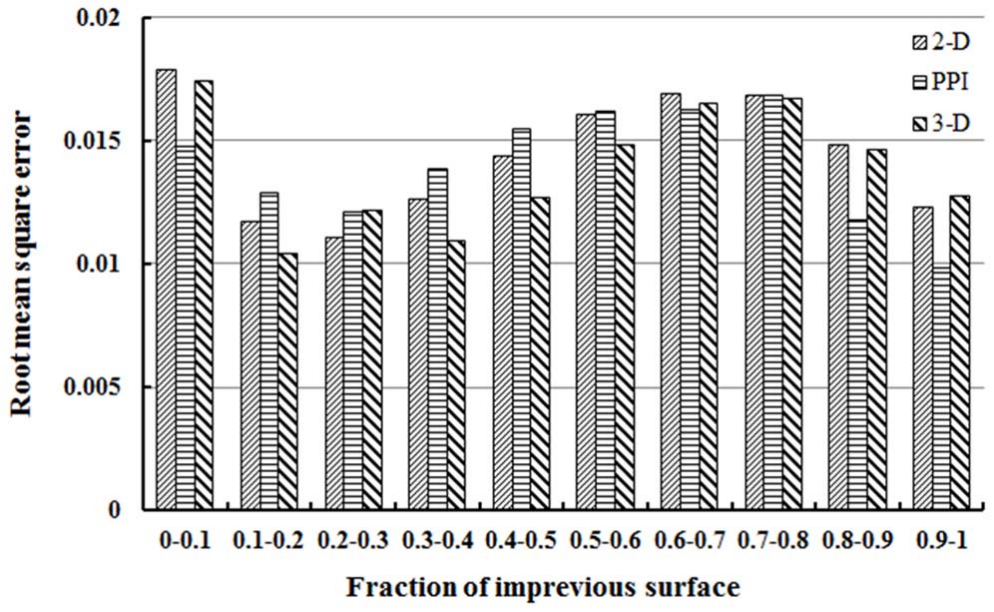
Error distribution along the value range of impervious surface fraction maps generated using different endmember derivation methods.

To provide some insight into the error structure within the model results, the value range of the impervious surface fraction map based on each method was divided into ten intervals and charted against the *RMSE* distribution (Figure 13). It revealed that the error distribution for the 3-D method basically followed a high-low-high-low pattern along with the increase of impervious fraction values. The highest error is located in the least impervious region (0.0–0.1), and the lowest in the second least region (0.1–0.2). It seemed to imply that the low-density and very high-density residential were hardest to characterize, whereas the medium-density residential and commercial land use types were easier to depict by the new method. Comparatively across all three methods, the 3-D scatter plot performed the best between 0.1 and 0.2 as well as 0.3 and 0.6, yet worse than PPI in both the low end (fraction <0.1) and the high end (fraction >0.8) of the range. In the other fraction ranges, the 3-D method showed no advantage over the other two methods.

**Figure 14:**
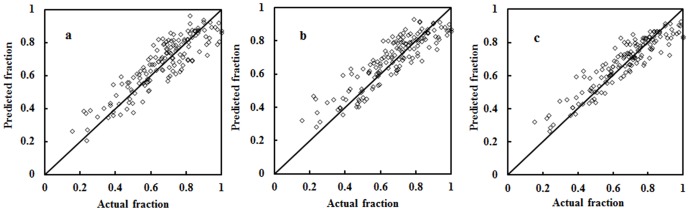
Comparisons of impervious surface estimation accuracy with endmembers derived from 2-D scatter plots (a), PPI (b), and 3-D scatter plot (c).

The second perspective of model evaluation involved comparing model results against the reference data obtained from color-infrared aerial photographs (see section 3.4 for details). The visual interpretation task resulted in 150 reference points selected through a stratified random sampling, each having a fraction value representing the percent impervious spectra within a 90×90 m^2^ area (i.e. the 3×3 ETM+ pixels). Fraction values from both sources were compared using a correlation analysis (Figure 14), and it was readily seen that a linear and positive relationship existed between the reference fractions and predicted ones for each of the three methods, indicating a high level of similarity between the model output and the reference data. For a more precise comparison between methods, numerical indices of *R^2^*, *MAE* and *RMSE* were computed and documented in Table 1. In this round of comparison, the 3-D method seemed to perform much better than the other two methods in all three error indices. Specifically, results from the 3-D method improved 6.5% over the 2-D method in *R^2^*, 18.5% in *MAE*, and 10.8% in *RMSE*, respectively. However, all the models seemed to follow a common tendency: low impervious surface fractions (<0.4) were overestimated; whereas high impervious surface fractions (>0.8) were underestimated. This caused the data fitting line to have a slope smaller than expected. On the other hand, such knowledge might be utilized to rectify the gains and biases of the models for better prediction.

**Table 1:** A comparison of model performance for impervious surface estimation.

Method	*R^2^*	*MAE*	*RMSE*
Endmembers derived from 2-D scatter plots	0.798	0.070	0.083
Endmembers derived from PPI	0.782	0.070	0.087
Endmembers derived from 3-D scatter plot	0.850	0.057	0.074

**Figure 15:**
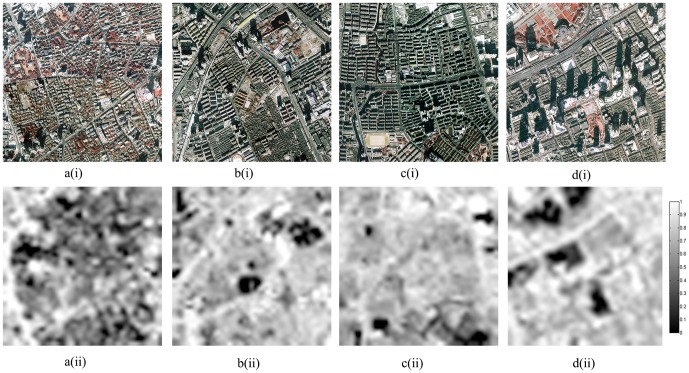
Spatial patterns of four typical land use types in Shanghai (color-infrared aerial photographs (above) and impervious surface fraction maps (below)) (a) medium-intensity residential; (b) high-intensity residential; (c) very high-intensity residential; (d) commercial.

From a qualitative perspective, fraction maps of four selected regions of different land use types (i.e. medium-residential, high-residential, very-high-density residential, and commercial) were further analyzed. These maps were visually compared with their corresponding areas on the color-infrared aerial photographs (Figure 15). All four cases in Figure 15 indicated that the distribution of estimated impervious surface seemed to follow the overall spatial pattern of the urban built structure in these regions. High fraction values appeared to coincide well with major transportation routes, large-size buildings, and large patches of cement pavement, typically comprising the urban structure of commercial and very-high-density land use types. Areas of medium brightness levels mixed with dark textural patterns in the fraction maps signified the mixture of urban built structures and vegetation to various degrees, a rather accurate characterization of the tree-covered, medium-density residential areas (Figure 15a) but less so for the high-density residential district (Figure 15b).

Despite the high visual agreement of spatial patterns between the reference data and estimated impervious surface fractions, some obvious exceptions can be found in commercial districts (Figure 15d), where there are large parcels very low in impervious surface fraction (Figure 15d(ii)) corresponding to yet apparent high-density residential areas on the aerial photographs (red rectangle in Figure 15d(i)). This situation was caused by the actual land use change occurring during the period between the acquisition times of observation data and reference data, as the very high-density residential land use in the aerial photographs (acquired in 2000) were converted to vegetated landscape one year later (captured by the Landsat data acquired in 2001). This marked change was further confirmed by additional aerial photographs acquired in 2002 (not shown in the article).

## Discussion

The comparative analysis presented above provides several important observations about the issue of model-to-method mismatch inherent in current practices of urban surface decomposition from remotely sensed data. While Ridd's triad model has been widely accepted as a fundamental working concept for urban surface decomposition, it is difficult to match the diverse spectra of the urban built environment to exact three components in practice. The spectral characteristics of our study region, Shanghai, again proved the compelling need for methodological changes to alleviate such mismatch, which was particularly outstanding when medium-resolution remote sensing imagery such as ETM+ data was used. Specifically, the dual spectral nature of urban impervious surface gave rise to the adoption of the four-component V-H-L-S conceptual framework and the development of the tetrahedron-based endmember selection approach to implement the framework. As evaluated by three quantitative error indicators (i.e. *R^2^*, *MAE*, and *RMSE*), this modification has led to overall accuracy improvement for impervious surface extraction, ranging from 6.5% to 10.8%, over the traditional 2-D scatter plots approach.

While the initial goal of designing the tetrahedron approach was achieved, this study actually found that both models could produce comparable and satisfactory results in urban surface decomposition. This observation is supported by the low model unmixing *RMSE* for both (0.014 and 0.016), similar *RMSE* structures for impervious surface endmember, and the small difference (6.5%) in the results of correlation analysis against reference data. This observation can be rationalized from two aspects. First, although the V-I-S model has been facing the conceptual challenge of spectral diversity in the component of impervious surface, this problem is much less significant in the actual realization of the decomposing procedure. Despite of the triad design, the split of impervious surface into high albedo and low albedo endmembers is a common practice, as indicated by many studies using the 2-D scatter plots and PPI endmember selection approaches [7], [17], [19], [33]. On the other hand, since the endmember selection process is highly interactive and thus necessarily subjective, it is always possible for an experienced analyst to find a set of pure pixels to properly represent the true endmembers in the scene, typically after many rounds of trial-and-error. In this process, cross-referencing multiple MNF 2-D scatter plots may achieve results equivalent to what the 3-D scatter plot can provide.

The very advantage of using the tetrahedron-based 3-D scatter plot lies in the fact that the visualization geometry perfectly matches the four components of urban surface that are modeled by the V-H-L-S conceptual framework. The four endmember model is optimal for Landsat TM and ETM+ imagery, and tetrahedron is the “smallest” simplex containing the data as the proxy for the “best” simplex in a 3-D space [46]. As hereby demonstrated, the naturally formed cloud of urban pixels under examination in a 3-D MNF space often resembles a solid of tetrahedron, which can be geometrically adjusted to fit the spectral data for the purpose of endmember visualization and selection. This has led to a more structured and semi-automated procedure for endmember extraction in this study, which greatly reduced the level of arbitrariness and human subjectivity in the pure pixel selection process and in turn facilitated quality extraction of urban endmembers. Based on the results presented in the previous section, the 3-D scatter plot approach seemed to have great potential for improving endmember selection.

In the 3-D scatter plot design, the major factors that have the direct and most important impact on endmember quality are the determination of an appropriate tetrahedron and the generation of sub-tetrahedrons. The critical step of tetrahedral demarcation is the search for an optimal set of four vertices, and the use of a genetic algorithm guided by a multi-objective function (i.e. MOGA) in the study seemed to handle it rather well. Unlike the N-FINDR algorithm by Winter [29], which adopts a fitness function based only on the maximum tetrahedral volume, the design of MOGA hereby considered both the minimum volume of simplex and the maximum number of pixels. One advantage of this design is being highly robust to noise in data, as data outliers are effectively kept away from the compact data cloud and not involved in endmember selection. In contrast, N-FINDR algorithm may be significantly affected by the image noise and provides a poor result (not show in the paper). On the other hand, since an endmember is generally comprised of a group of pixels with similar spectra, identifying these pixels becomes the task of sub-tetrahedron determination. This step involved an incremental testing procedure to locate the base of the sub tetrahedron, which was made to converge when spectral characteristics of the current pixel set and previous one are virtually significant difference. Although this seemingly automatable process was conducted in an interactive fashion in the study, it produced quality endmembers that helped to improve imperviousness fraction mapping.

## Conclusions

Quality decomposition of an urban surface from remotely sensed data largely depends on its representing conceptual model and model implementing methodology. This study thus aimed to enhance the adaptability of the classic V-I-S model for urban surface mapping by expanding it into a tetrahedron framework. Along with the model modification was a quadrangular design of tetrahedral geometry to match the spectral data distribution in a 3-D feature (or MNF) space. Compared to the traditional 2-D scatter plots and PPI methods, the new approach produced favorable results for urban imperviousness characterization in Shanghai, China. As demonstrated in the empirical study, this approach seemed to have two major benefits. The first benefit is that viewing and selection of pure pixels become more intuitive and easier due to the integration of multiple views of spectral data into one three-dimensional view, so that both commission and omission errors can be minimized. The second benefit is that the tetrahedron design facilitated automation of the endmember extraction process, such as MOGA with the fitness function in this study, making it less rely on human interference. Although the sub-tetrahedron determination was still conducted interactively, the potential of automating the process is great owing to its iterative nature and quantitative converging criteria.

The preliminary results from this study also revealed some areas for model and method improvement. At the technical level, for example, the stability and robustness of the endmember selection method need to be further tested using different study areas or different datasets. In an even broader context, some important questions also need to be addressed, including whether the new model/method can consistently yield better results than traditional methods, and what if the distribution of spectral data in the 3-D feature space shows up as a polyhedron with more than four vertices. The second case is usually related to the processing of high-resolution remote sensing data that contain intricate spectral characteristics for each urban land cover type. All these apparently require a more automated and self-adaptive approach to endmember modeling for accurate urban surface mapping.
